# Evaluating the relationship between pain empathy, knowledge and attitudes among nurses in North China: a cross-sectional study

**DOI:** 10.1186/s12912-023-01577-2

**Published:** 2023-10-31

**Authors:** Lihua Wu, Xingyu Chen, Shaofen Jia, Liya Yan, Jia Li, Liwei Zhang, Yanjing Guo, Jingjing Lu, Wanling Li

**Affiliations:** 1 Lymphatic Oncology Department, Shanxi Bethune Hospital, Taiyuan, Shanxi China; 2 Oncology Center, Shanxi Bethune Hospital, Taiyuan, Shanxi China; 3 School of Nursing, Shanxi University of Traditional Chinese Medicine, Taiyuan, Shanxi China; 4 Department of Geriatrics, Tongji Hospital, Tongji Medical College, Huazhong University of Science and Technology, Wuhan City, Hubei Province China; 5 Nursing department, Shanxi Bethune Hospital, Taiyuan, Shanxi, China

**Keywords:** Pain, Nurses, Knowledge, Attitudes, Empathy

## Abstract

**Background:**

Effective pain management is closely related to nurses’ knowledge, attitudes and empathy regarding pain. Nursing educators and managers should understand the relationship between nurses’ pain management knowledge, attitudes and empathy level, and take targeted measures accordingly. Currently, there is limited study exploring the relationship between pain empathy and pain knowledge and attitudes among nurses in North China.

**Objectives:**

The purpose of this study was to investigate the level of nurses’ pain management knowledge and attitudes and pain empathy, to analyze the factors influencing pain empathy, and to explore the relationship between these two variables.

**Design:**

This study was a quantitative, descriptive-correlation design.

**Setting and participants:**

The study population was registered nurses in North China, the sample included 177 registered nurses in North China.

**Methods:**

Data were collected with the “General data questionnaire”, “Knowledge and attitudes survey regarding pain” (KASRP) and the “Empathy for pain scale” (EPS) via Wechat mini program “Questionnaire Star”.

**Results:**

The 177 registered nurses completed the survey. The averege correct rate for KASRP was (51.94 ± 9.44)%, and none of the respondents achieved a percentage score of >80%. The mean score for pain empathy was (2.78 ± 0.78), the empathy reactions dimension was (2.99 ± 0.77), and the body and mind discomfort dimension was (2.71 ± 0.80). The results of multiple stepwise linear regression showed that whether they had received empathy training, whether they had greater trauma or severe pain and whether they had negative emotions were independent influencing factors for EPS scores. Pearson correlation analysis showed that KASRP scores were positively correlated with EPS scores (r = 0.242, P < 0.05).

**Conclusions:**

The pain knowledge and attitudes of nurses in North China are far from optimal. Nurses have a relatively low accuracy rate in areas such as medication knowledge, assessment of patient pain based on case studies, and handling PRN prescriptions. Nursing educators and administrators need to design some pain management courses in a targeted manner. Nurses’ empathy for pain was at a moderate level. Pain empathy was positively correlated with pain knowledge and attitudes, suggesting that empathy for pain can be developed postnatally.

## Introduction

In 2020 the International Association for the Study of Pain(IASP) redefined pain as “an unpleasant sensory and emotional experience associated with, or resembling that associated with, actual or potential tissue damage” [1]. Survey findings the prevalence of chronic pain was 23.7% among Iranian adolescents [2]. An estimated 20.4% of U.S. adults (50.0 million) had chronic pain and the prevalence of chronic pain increases with age [3, 4]. Despite today’s medical advances and improvements, inadequate pain management remains a global problem [5]. Inadequate pain management can have many effects on patients, such as prolonging hospital stays [6], causing psychological problems such as anxiety and depression [7], reducing work participation, affecting daily life, posing a financial burden on the patient and healthcare system [8–10].

Pain management is defined as the process of improving pain through the assessment, documentation, treatment and care of pain, leading to controlling pain, improving comfort and quality of life of patients [11]. Nurses have a crucial role in pain management as pain assessors, implementers of pharmacologic and nonpharmacologic analgesic measures, and educators of patients and families about analgesia. It is evident that nurses have adequate knowledge of pain management and a positive attitude are essential for effective pain management [12, 13]. Therefore, only by understanding which aspects of nurses’ pain management knowledge are weak and lacking can we provide targeted education and training based on this.

In addition to knowledge and attitude, empathy is considered an indispensable skill for pain management [14, 15]. Empathy is the ability to put oneself in the shoes of others and understand their situations and feelings [16, 17]. Empathy enables patients to feel understood and respected and promotes self-expression. At the same time, empathy enables health care professionals to correctly understand patients’ feelings, to assess patients more comprehensively and accurately, and to improve their diagnosis and sense of professional value [18–20]. Therefore, in order to assess pain effectively, nurses should correctly understand patients’ feelings, thoughts and reactions, which also requires nurses to acquire empathic knowledge and skills.

Pain empathy is one of the more typical manifestations of empathy, which refers to an individual’s perception, judgment and emotional response to another person’s pain when he or she perceives the pain or injury state of the other person, and is a pro-social behavior that includes both emotional and cognitive aspects [21]. Acqua C et al. monitored nurses’ pain management behaviors in the emergency department for 15 months and found that brain signals recorded when empathizing with others predicted how often nurses recorded patient pain, and that health care providers who were not sensitive to patient pain reported less patient pain when empathic behaviors did not occur [22]. Pain empathy not only motivates individuals to perceive the pain of others, generating empathy and maintaining good interpersonal relationships; it also helps individuals to stay alert and avoid possible dangers [23, 24]. Clinical nurses are important assessors of patient pain, implementers of analgesic measures and health education throughout the pain management process. For nurses, pain empathy can help them obtain more information about patients with pain, as well as improve pain patients’ active participation in health care, and indirectly influence the accuracy of pain assessment and alleviate patients’ fears due to pain, so it is clinically important to understand their pain empathy and the factors influencing it.

Studies have found that medical professionals have significantly lower levels of empathy than non-medical professionals in clinical settings, which may be related to professional knowledge and personal experience [25]. The level of empathy among medical students after medical training was overall decreased, but was increased in terms of valuable empathy in positive doctor-patient interactions [26]. However, it has also been found that although the level of empathy and pain knowledge and attitudes of student nurses were weakly positively correlated, this was not statistically significant [27]. Currently, there are few studies on pain empathy among nurses in China, and the correlation between pain management knowledge and attitudes and pain empathy and the factors influencing pain empathy are unclear. Therefore, further research is needed to explore the correlation between nurses’ pain management knowledge and attitudes and pain empathy.

The purpose of the present study was to investigate the level of nurses’ knowledge and attitudes toward pain management and to explore the relationship between the level of empathy and pain knowledge and attitudes. The research questions include: (1) weaknesses in nurses’ pain knowledge and attitudes in North China; (2) nurses’ pain empathy levels and the factors influencing them, and explore ways to improve individual pain empathy; (3) correlation analysis between nurses’ pain empathy levels and pain management knowledge and attitudes.

## Methods

The research project was in accordance with the principles of the Declaration of Helsinki regarding medical research in humans, following local regulations. This study was approved by the Medical Ethical Committee of Shanxi Bethune Hospital (YXLL-2023-071)and informed consent was obtained from the participants.

### Study design and setting

This was a quantitative, descriptive correlation study. The cross-sectional survey was conducted using the WeChat mini program “Questionnaire Study”, which involved nursing staff working in Shanxi Province between Feb 10 and Apr 10, 2023.

### Study population and sampling

A snowball sampling method was used to recruit nursing staff through the WeChat mini program “Questionnaire Star”. In this process, the nurses who worked in the Oncology Center of Shanxi Bethune Hospital were initially invited to complete the questionnaire. In addition, other nurses from a total of 10 hospitals were recruited from the invitation of initial respondents via WeChat contacts. To be eligible for the survey, nurses had to be (1) registered nurses engaged in clinical work, and (2) working for ≥ 1 year, and (3) informed and voluntary participation. Exclusion criteria: (1)practical nurses or resigned nurses, or (2) administrative nurses who were not directly involved in the clinical care of patients, or (3) those who dropped out.

After deleting the multiple responses of same IP address and short completion time (under 300s), there were 177 valid questionnaire in total (response rate was 85.1%).

### Assessments

(I) Participant characteristics: A self-developed general data questionnaire was used to collect the participant characteristics, which included: hospital grade, gender, age, department, title, years of work, education level, marital status, etc.

(II) Pain knowledge and attitudes: We used the Knowledge and Attitudes Survey Regarding Pain (KASRP) to evaluate the pain management knowledge and attitudes level of nurses. The scale was developed by McCaffery and Betty in 1987 based on the pain management criteria proposed by the American Institute for Health Care Policy Research, WHO and the American Pain Society [28]. It was translated into multiple languages and was often used when assessing nurses’ knowledge and attitudes toward pain management. It was last revised in 2014. The revised questionnaire had good reliability and validity, and its Cronbach’s alpha was greater than 0.70. The Chinese version of the scale had shown a high internal consistency, with a Cronbach’s alpha of 0.717. The scale had 6 dimensions including pain assessment (8 items), medicine (24 items), intervention (3 items), addiction (3 items), spiritual/cultural (2 items) and pathophysiology (1 item) and 41 questions which included 22 true/false, 15 multiple choice and two case studies with two questions for each case. An item answered correctly was assigned 1 point, and 0 point for an item answered incorrectly or not. Total scores were summed ranging from 0 to 41. A minimum of 80% accuracy rate was considered to be qualified. In this study, the Cronbach’s alpha value of the scale was 0.705.

(III) Pain empathy: We used the Empathy for Pain Scale (EPS) developed by Giummarra [21] to assess pain empathy of nurses. The scale had 48 items and 2 dimensions including the empathy reactions and body and mind discomfort reactions. A 5-point Likert scale ranging from 0 (‘totally disagree’) to 5 (‘totally agree’) was used to score all items. The total score of the scale was the average score of each item, which was greater than 3 points indicating a high level of individual pain empathy. The Cronbach’s alpha of the Chinese version scale was 0.914. Cronbach’s alpha of the scale in the present study was 0.968.

### Statistical analyses

Use SPSS 24.0 software for data analysis. Use frequency and percentage to describe demographic data. Use mean ± standard deviation to describe the answer rate of the survey on nurses’ pain management knowledge and attitudes and pain empathy scores. Use independent samples t-test or one way ANOVA to compare means for data that meets normal distribution. Use Mann-Whitney U test or Kruskal-Wallis H test to compare data that does not meet normal distribution.Use multiple linear regression analysis to identify factors that influence nurses’ pain empathy level. Use Pearson correlation test to analyze the correlation between KASRP and EPS scores. Statistical significance was set at < 0.05.

## Results

### Correct responses to the items in KASRP and scores

Table 1 showed the correct responses of the 177 nurses. The average correct rate for KASRP was (51.94 ± 9.44)%, only six questions with a correct rate greater than 80% and the highest answer rate was only 75.61%. Table 2 showed the total score and subscores of KASRP.

**Table 1:** Correct answers of nurses knowledge and attitudes regarding pain(N = 177)

Items	Correct(%)
1.Vital signs are always reliable indicators of the intensity of a patient’s pain.	53.11
2.Because their nervous system is underdeveloped, children under two years of age have decreased pain sensitivity and limited memory of painful experiences.	22.03
3.Patients who can be distracted from pain usually do not have severe pain.	50.28
4.Patients may sleep in spite of severe pain.	28.81
5.Aspirin and other nonsteroidal anti-inflammatory agents are NOT effective analgesics for painful bone metastases.	35.60
6.Respiratory depression rarely occurs in patients who have been receiving stable doses of opioids over a period of months.	49.15
7.Combining analgesics that work by different mechanisms (e.g., combining an NSAID with an opioid) may result in better pain control with fewer side effects than using a single analgesic agent.	79.10
8.The usual duration of analgesia of 1–2 mg morphine IV is 4–5 h.	28.81
9.Opioids should not be used in patients with a history of substance abuse.	35.03
10.Elderly patients cannot tolerate opioids for pain relief.	53.11
11.Patients should be encouraged to endure as much pain as possible before using an opioid.	75.71
12.Children less than 11 years old cannot reliably report pain so clinicians should rely solely on the parent’s assessment of the child’s pain intensity.	83.62
13.Patient’s spiritual beliefs may lead them to think pain and suffering are necessary.	74.01
14.After an initial dose of opioid analgesic is given, subsequent doses should be adjusted in accordance with the individual patient’s response.	92.66
15.Giving patients sterile water by injection (placebo) is a useful test to determine if the pain is real.	26.56
16.Vicodin (hydrocodone 5 mg + acetaminophen 300 mg) PO is approximately equal to 5–10 mg of morphine PO.	67.23
17.If the source of the patient’s pain is unknown, opioids should not be used during the pain evaluation period, as this could mask the ability to correctly diagnose the cause of pain.	11.86
18.Anticonvulsant drugs such as gabapentin (Neurontin) produce optimal pain relief after a single dose.	51.41
19.Benzodiazepines are not effective pain relievers and are rarely recommended as part of an analgesic regiment.	75.71
20.Narcotic/opioid addiction is defined as a chronic neurobiologic disease, characterized by behaviors that include one or more of the following: impaired control over drug use, compulsive use, continued use despite harm, and craving.	83.05
21.The term ‘equianalgesia’ means approximately equal analgesia and is used when referring to the doses of various analgesics that provide approximately the same amount of pain relief.	92.66
22.Sedation assessment is recommended during opioid pain management because excessive sedation precedes opioid-induced respiratory depression.	93.79
23.The recommended route of administration of opioid analgesics for patients with persistent cancer-related pain is:	45.76
24.The recommended route of administration of opioid analgesics for patients with brief, severe pain of sudden onset, such as trauma or postoperative pain is:	46.33
25.Which of the following analgesic medications is considered the drug of choice for the treatment of prolonged moderate to severe pain for cancer patients?	66.10
26.A 30 mg dose of oral morphine is approximately equivalent to:	54.24
27.Analgesics for post-operative pain should initially be given	71.19
28.A patient with persistent cancer pain has been receiving daily opioid analgesics for 2 months. Yesterday the patient was receiving morphine 200 mg/hour intravenously. Today he has been receiving 250 mg/hour intravenously. The likelihood of the patient developing clinically significant respiratory depression in the absence of new comorbidity is:	23.16
29.The most likely reason a patient with pain would request increased doses of pain medication is:	68.36
30.Which of the following is useful for treatment of cancer pain?	48.59
31.The most accurate judge of the intensity of the patient’s pain is:	71.19
32.Which of the following describes the best approach for cultural considerations in caring for patients in pain?	81.36
33.How likely is it that patients who develop pain already have an alcohol and/or drug abuse problem?	42.94
34.The time to peak effect for morphine given IV is:	70.62
35.The time to peak effect for morphine given orally is:	22.03
36.Following abrupt discontinuation of an opioid, physical dependence is manifested by the following:	14.69
37.Which statement is true regarding opioid induced respiratory depression?	32.20
38 A.Patient A: Andrew is 25 years old and this is his first day following abdominal surgery. As you enter his room, he smiles at you and continues talking and joking with his visitor. Your assessment reveals the following information: BP = 120/80; HR = 80; R = 18; on a scale of 0 to 10 (0 = no pain/discomfort, 10 = worst pain/discomfort) he rates his pain as 8. On the patient’s record you must mark his pain on the scale below. Circle the number that represents your assessment of Andrew’s pain.	2.26
38B.Your assessment, above, is made two hours after he received morphine 2 mg IV. Half hourly pain ratings following the injection ranged from 6 to 8 and he had no clinically significant respiratory depression, sedation, or other untoward side effects. He has identified 2/10 as an acceptable level of pain relief. His physician’s order for analgesia is “morphine IV 1–3 mg q1h PRN pain relief.” Check the action you will take at this time.	8.47
39 A.Patient B: Robert is 25 years old and this is his first day following abdominal surgery. As you enter his room, he is lying quietly in bed and grimaces as he turns in bed. Your assessment reveals the following information: BP = 120/80; HR = 80; R = 18; on a scale of 0 to 10 (0 = no pain/discomfort, 10 = worst pain/discomfort) he rates his pain as 8. On the patient’s record you must mark his pain on the scale below. Circle the number that represents your assessment of Robert’s pain:	8.47
39B.Your assessment, above, is made two hours after he received morphine 2 mg IV. Half hourly pain ratings following the injection ranged from 6 to 8 and he had no clinically significant respiratory depression, sedation, or other untoward side effects. He has identified 2/10 as an acceptable level of pain relief. His physician’s order for analgesia is “morphine IV 1–3 mg q1h PRN pain relief.” Check the action you will take at this time:	19.78

**Table 2:** KASRP scores (N = 177, M ± SD)

Variables	Number of items	Scores
Pain assessment	8	3.51 ± 1.37
Medicine	24	14.01 ± 2.87
Intervention	3	0.75 ± 0.68
Addiction	3	1.41 ± 0.68
Spiritual/cultural	2	1.38 ± 0.64
Pathophysiology	1	0.22 ± 0.42
Total questionnaire	41	21.29 ± 3.87

### Empathy for pain scale scores

The mean score of the nurses’ pain empathy was (2.78 ± 0.78), with a mean score of (2.99 ± 0.77) for the empathy reactions dimension and a mean score of (2.71 ± 0.80) for the body and mind discomfort reactions dimension(Table 3).

**Table 3:** Nurses Pain Empathy Scores(N = 177)

Variables	Mean score	Minimum score	Maximum score
Empathy for pain	2.78 ± 0.78	1.46	5.00
Empathy reactions	2.99 ± 0.77	1.67	5.00
Body and mind discomfort reactions	2.71 ± 0.80	1.36	5.00

### Characteristics and pain empathy scores of nurses with different characteristics of nurses

A total of 177 nurses were investigated in this study. Participant characteristics and pain empathy scores of nurses with different characteristics of nurses are presented in Table 4. Statistically significant differences in pain empathy scores among nurses with different ages, professional titles, years of work experience, marital status, whether they had received empathy training, whether they were only children, whether they had experienced greater trauma or severe pain, whether they had negative emotions, and whether they had experience working in oncology (P<0.05)(Table 4).

**Table 4:** Sociodemographic and pain-related characteristics and pain empathy scores of nurses with different characteristics of nurses(N = 177)

Variables	Frequency(Percentage) N(%)	Score(M ± SD)	Statistical Quantity	P Value
Hospital grade				
Tertiary hospital	157(88.70)	2.76 ± 0.76	0.836^a^	0.404
Below the Tertiary hospital	20(11.30)	2.92 ± 0.94		
Gender				
Male	8(4.52)	2.76 ± 0.60	0.080^a^	0.936
Female	169(95.48)	2.78 ± 0.79		
Age(years)				
20~	53(29.94)	2.61 ± 0.72	4.166^b^	0.007
26~	51(28.81)	2.68 ± 0.68		
31~	54(30.51)	2.87 ± 0.84		
36~	19(10.74)	3.28 ± 0.81		
Department				
Oncology	85(48.02)	2.87 ± 0.67	1.567^b^	0.172
Surgical	30(16.95)	2.69 ± 0.79		
Internal medicine	25(14.13)	2.77 ± 1.00		
Pediatrics	9(5.08)	3.18 ± 1.05		
Obstetrics and gynecology	9(5.08)	2.52 ± 0.72		
Other units	19(10.74)	2.48 ± 0.71		
Professional title				
Primary care nurse	120(67.80)	2.67 ± 0.76	3.757^b^	0.025
Intermediate nurse	52(29.38)	2.98 ± 0.80		
Senior nurse	5(2.82)	3.23 ± 0.48		
Years of nursing experience				
<5	91(51.41)	2.62 ± 0.73	3.848^b^	0.023
5 ~ 10	35(19.78)	2.92 ± 0.77		
>10	51(28.81)	2.96 ± 0.83		
Educational level				
College or below	16(9.04)	2.96 ± 0.87	0.465^b^	0.481
university	155(87.57)	2.76 ± 0.78		
master or below	6(3.39)	2.72 ± 0.61		
Number of night shifts/month				
0 ~ 2	47(26.56)	2.89 ± 0.62	0.734^b^	0.481
3 ~ 5	67(37.85)	2.77 ± 0.83		
≥ 6	63(35.59)	2.71 ± 0.83		
Marital Status				
Not married	88(49.72)	2.59 ± 0.71	3.244^a^	0.001
Married	89(50.28)	2.96 ± 0.80		
Whether trained in empathy				
Yes	64(36.16)	2.99 ± 0.78	2.825^a^	0.005
No	113(63.84)	2.66 ± 0.75		
Whether only child				
Yes	34(19.21)	2.54 ± 0.67	2.040^a^	0.043
No	143(80.79)	2.84 ± 0.79		
Personality traits				
Introvert	39(22.03)	2.80 ± 0.77	0.048^b^	0.953
Middle	102(57.63)	2.78 ± 0.74		
Extrovert	36(20.34)	2.74 ± 0.89		
Whether experienced major trauma or severe pain				
Yes	50(28.25)	3.19 ± 0.81	4.649^a^	<0.001
No	127(71.75)	2.62 ± 0.70		
Self-perceived pain tolerance level				
Very poor	9(5.08)	2.67 ± 0.46	0.628^b^	0.643
Relatively poor	23(12.99)	2.66 ± 0.76		
Average	94(53.11)	2.74 ± 0.75		
Relatively good	48(27.13)	2.91 ± 0.90		
Very good	3(1.69)	2.99 ± 0.34		
Job satisfaction				
Very dissatisfied	2(1.13)	2.46 ± 0.44	0.852^b^	0.494
Relatively dissatisfied	66(37.29)	2.80 ± 0.80		
Average	61(34.46)	2.75 ± 0.69		
Relatively satisfied	33(18.64)	2.67 ± 0.67		
Very satisfied	15(8.48)	3.08 ± 1.20		
Negative emotion				
Yes	93(52.54)	2.66 ± 0.81	2.213^a^	0.028
No	84(47.46)	2.91 ± 0.73		
Work experience of oncology				
Yes	100(56.50)	2.89 ± 0.76	2.231^a^	0.027
No	77(43.50)	2.63 ± 0.78		

^a^: t-test, ^b^: one way ANOVA

### Multiple stepwise liner regression analysis of EPS scores

Using the nurse pain empathy score as the dependent variable and the variables that were statistically significant in the univariate analysis as independent variables in the regression equation (see Table 5 for assignment), nurses who had received empathy training, experienced trauma or severe pain, and did not have negative emotions were more likely to have high levels of pain empathy (all P < 0.05), as shown in Table 6.

**Table 5:** Assignment of Independent Variables

Independent Variable	Assignment Method
EPS scores	EPS scores
Age	20 ~ = 1, 26 ~ = 2, 31 ~ = 3, 36 = 4
Professional title	Primary care nurse = 1, Intermediate nurse = 2, Senior nurse = 3
Years of nursing experience	<5 = 1, 5 ~ 10 = 2, >10 = 3
Marital Status	Not married = 1, Married = 2
Whether trained in empathy	Yes = 1, No = 2
Whether only child	Yes = 1, No = 2
Whether experienced major trauma or severe pain	Yes = 1, No = 2
Negative emotion	Yes = 1, No = 2
Work experience of oncology	Yes = 1, No = 2

**Table 6:** Multiple Stepwise Regression Analysis of Nurses’ empathy for pain(N = 177)

Variable	Regression Coefficient	S.E.	Standardized Regression Coefficient	t Value	p Value
Constant term	3.033	0.530		5.721	0.000
Age	0.137	0.081	0.175	1.694	0.092
Professional title	0.158	0.131	0.109	1.210	0.228
Years of nursing experience	-0.169	0.119	-0.189	-1.421	0.157
Marital Status	0.155	0.171	0.100	0.903	0.368
Whether trained in empathy	-0.246	0.116	-0.152	-2.124	0.035
Whether only child	0.175	0.144	0.089	1.216	0.226
Whether experienced major trauma or severe pain	-0.485	0.126	-0.281	-3.847	<0.001
Negative emotion	0.248	0.108	0.160	2.300	0.023
Work experience of oncology	-0.104	0.113	-0.067	-0.922	0.358

F = 5.446, P<0.001; determination coefficient R^2^ = 0.227, adjusted R^2^ = 0.185

### Correlation analysis of empathy for pain and knowledge and attitudes regarding pain

To assess the strength of linearity between pain empathy and pain knowledge and attitudes, we performed a Pearson correlation analysis between the two variables. The results of the Pearson correlation analysis showed a positive correlation between pain empathy and pain knowledge and attitudes (r = 0.242, P < 0.05), as shown in Table 7.

**Table 7:** Correlation of nurse pain empathy with pain knowledge and attitudes(r, N = 177)

Variables	Empathy for pain	
	r value	p value
Knowledge and attitudes regarding pain	0.242	0.001

## Discussion

The results of this study showed that the nurses’ knowledge and attitudes toward pain management was (51.94 ± 9.44)%, which was lower than the original authors’ standard of “80% or more of the answers were passing”, and it is noteworthy that only six questions with a correct rate greater than 80% and the highest answer rate was only 75.61%, which means that no nurses in this survey reached the passing standard, indicating that the knowledge and attitudes of nurses about pain management in northern China are far from optimal. In this study, the level of pain management knowledge and attitudes of nurses in northern China (51.94 ± 9.44)% was higher than the average level of nurses in low-income areas of China (40.3 ± 7.95)% investigated by Qu et al. [29], which may be explained by the relatively poor learning resources and learning opportunities for nurses in economically underdeveloped areas. This also suggests that better-equipped tertiary hospitals should make full use of online resources when conducting pain management training and share learning resources with hospitals in poorer areas.

In this study, the level of pain management knowledge and attitudes of nurses in northern China was lower than that of nurses in countries such as the United States [30] and Spain [31]. The reason may be that there are more issues about medications such as isodose conversion of pain medications and side effects of pain medications in KASRP, while in China nurses do not have prescribing authority, and pain management work for patients only includes pain assessment, implementation of pain relief measures as prescribed, and determination of drug addiction, and rarely involves such tasks as isodose conversion of pain medications and observation of side effects such as respiratory depression. In 2021, the International Council of Nurses (ICN) released the world’s first “Guidelines for Nurses’ Prescribing Rights” [32], recognizing the important role of nurses in safeguarding global health and launching the Global Initiative for Nurses’ Prescribing Rights, which shows that granting nurses prescribing rights has become a global trend [33, 34]. Although nurse prescribing authority is still not legalized in most areas of China, with the increasing aging of China and the growing incidence of chronic diseases, the existing medical care can no longer fully meet people’s health needs, and it is an effective strategy to alleviate the relative shortage of medical resources by granting nurses the right to prescribe drugs [35, 36]. Of course, this also places a higher demand on Chinese nurses’ knowledge of medications. Therefore, hospitals should design pain management curricula with a targeted focus on knowledge related to pain medications.

The results of this study showed that the correct rate of the question“The most accurate judge of the intensity of the patient’s pain is the patient” was 71.19%, but the correct rate of the assessment of the patient’s pain in the case (questions “38A” and “39A” was only 2.26% and 8.47%, which was the lowest two questions. This indicates that most nurses know that the patient’s complaints are the gold standard for pain assessment, but when analyzing specific cases, nurses are disturbed by the patient’s expressions and vital signs, resulting in inaccurate assessments. This finding was supported by other studies in the literature [37, 38]. The study found that teacher-led, lecture-based, knowledge-only teaching methods often resulted in poor training results due to repetitive learning content and failure to increase nurses’ interest in learning [39]. High simulation training and situational simulation are learner-centered. By setting up reasonable situational problems and simulated practice, nurses can effectively use the difficulties they feel in real situations to stimulate a sense of conquest and interest in learning, which is conducive to improving learning participation and understanding of learning content, as well as combining theory and practice, transforming theoretical knowledge into clinical skills, and truly improving clinical nursing competence [40–42]. Therefore, when designing the curriculum of pain management, school and hospitals should incorporate appropriate theoretical frameworks such as Nursing Simulation Teaching Theory [43], along with case studies and simulated real situations to enhance nurses’ interest in learning, improve training effectiveness, so as to really improve nurses’ pain management knowledge and attitudes.

PRN is defined as a medical prescription that is administered according to the immediate needs of patients, rather than at a predetermined time of administration [44]. Analgesics are one of the common PRN medications prescribed. The results of this study showed that the two multiple-choice questions on the intervention dimension (from two case studies of abdominal surgery in which nurses had to make decisions about the patient’s medication based on the patient’s pain level and PRN description) were among the six questions with the lowest correct rates. This was consistent with the findings of Ortiz et al. [37]. These two problems were related to the nurses’ decision to give PRN opioids (do not give morphine or give morphine 1, 2, or 3 mg) after assessing the patient’s pain level. This indicates that the vast majority of nurses are unable to properly handle PRN orders regarding pain. The reason for this may be that nurses do not know enough about the side effects of pain medication and are concerned that overdosing may cause addiction to the patient. Although there are many benefits to PRN medication management, such as empowering nurses and patients, and providing flexibility in relieving patients’ physical and emotional distress [45], it is a complex task for nurses to make decisions about PRN medications, and errors in handling PRN orders do occur, which can lead to problems such as overdose or underdose of medications [46]. In addition, the study found that most PRN medical orders did not describe effects and adverse effects after treatment [44]. Therefore, care managers or educators should specifically add the content on proper management of PRN orders to their pain management training and develop specific systems regarding what must be documented after PRN orders are executed.

In this study, the total pain empathy score of nurses was (2.78 ± 0.78), which was slightly lower than the EPS with a score of 3 as a criterion for high level of pain empathy, indicating that the pain empathy of nurses in this group was at a moderate level. The empathy response dimension was (2.99 ± 0.77), suggesting that nurses’ pain empathy reactions was at a high level, which could promote empathy and concern for patients and other empathic responses, and was conducive to the establishment of a good nurse-patient relationship. The score of body and mind discomfort reactions dimension was (2.71 ± 0.80), suggesting a moderate level of body and mind discomfort reactions in this group of nurses. Although pain empathy can help nurses better understand patients’ feelings and maintain a good doctor-patient relationship, studies [47, 48] have found that health care workers can be highly susceptible to inducing their own somatic discomfort and even empathy fatigue by observing patients’ pain and processing their own previous similar pain experiences, reducing their work efficiency. Therefore, managers should pay attention to the importance of nurses’ empathic response to pain in nursing, and they also need to be alert to the negative effects of pain empathy on nurses themselves.

The results of this study showed that nurses who received empathy training had higher levels of pain empathy. It is suggested that empathy training is an effective method to improve nurses’ pain empathy level. However, it is important to note that when designing empathy courses, it is not advisable to focus solely on how to improve pain empathy skills. Instead, methods for avoiding excessive physical and mental discomfort caused by pain empathy should also be increased. Additionally, the results of this study showed that nurses who have had previous experiences with pain had higher levels of pain empathy. This is consistent with the findings of Wang et al. [49, 50] who found that patients with menstrual pain had higher levels of pain empathy than healthy controls. This may be because nurses who have experienced pain themselves are better able to engage in “mentalizing” or imagining themselves in another person’s position, and have stronger abilities to perceive others’ pain, which facilitates empathy with others [51, 52]. But there are also studies that have come to the opposite conclusion - patients who suffer from chronic pain show lower levels of perspective-taking and empathetic concern [53, 54]. Thus, the observers’ own pain experience was associated with pain empathy, but the exact relationship is unclear and further research is needed in the future to explore this relationship and the mechanisms behind it. In addition, the results of this study also showed that nurses with higher levels of negative emotionality had lower levels of pain empathy. Li et al. [55]found that the negative mood suppresses the motoric empathic resonance for others’ pain. This suggests that the level of pain empathy is related to emotional state. It suggests that managers should pay more attention to nurses’ negative emotions and actively take measures such as mindfulness intervention [56] and resilience education programme [57] etc. eliminating nurses’ negative emotions to help nurses improve their pain empathy.

The results of this study showed a positive correlation between pain empathy and pain knowledge and attitudes. Nurses with higher levels of empathy may be more likely to understand patients’ pain experiences, which in turn helps them assess and manage patients’ pain. Also, nurses with higher levels of pain management knowledge and attitudes are more empathetic to the patient’s experience of pain. This also suggests that pain empathy can be developed later in life. However, there are also studies that have reached the opposite conclusion [25, 26]. Therefore, more research is needed to explore their relationship in the future. The above findings also provide a theoretical basis for developing intervention strategies to improve nurses’ pain empathy.

### Limitations

There are some limitations in this study. First, Due to the time and financial constraints, this study only surveyed hospitals in Shanxi Province and did not include nurses from other regions. As a result, the study subjects may lack representativeness. Thus the findings of this study may not be generalizable to other nurses. Subsequent research can expand the scope of the investigation. Second, this study explored the influencing factors of pain empathy levels, but there may be incomplete influencing factors included. Subsequent studies may explore more comprehensive potential influencing factors in depth to provide a strong reference for constructing pain empathy intervention strategies for nurses. Third, as this is a cross-sectional study, it was not possible to conclude a causal relationship between empathy for pain and pain knowledge and attitudes.

## Conclusions

In summary, the current study indicates that the pain knowledge and attitudes of nurses in North China are far from optimal. Nurses have a relatively low accuracy rate in areas such as medication knowledge, assessment of patient pain based on case studies, and handling PRN prescriptions. Nursing educators and administrators need to design some pain management courses in a targeted manner. Nurses’ empathy for pain was at a moderate level. Pain empathy was positively correlated with pain knowledge and attitudes, suggesting that empathy for pain can be developed postnatally.

## Data Availability

The datasets used during the current study are available from the corresponding author on reasonable request.
